# Integrated 3D printing solution to mitigate shortages of airway consumables and personal protective equipment during the COVID-19 pandemic

**DOI:** 10.1186/s12913-020-05891-2

**Published:** 2020-11-12

**Authors:** Ferran Fillat-Gomà, Sergi Coderch-Navarro, Laia Martínez-Carreres, Núria Monill-Raya, Toni Nadal-Mir, Cristina Lalmolda, Manel Luján, Candelaria de Haro, Lluís Blanch

**Affiliations:** 1 3D Surgical Planning Lab. Parc Taulí Hospital Universitari. Institut d’Investigació i Innovació Parc Taulí (I3PT), Universitat Autònoma de Barcelona, Par Taulí 1. Santa Fe Building 2nd floor, 08208 Sabadell, Spain; 2 Department of Orthopaedic Surgery and Traumatology. Parc Taulí Hospital Universitari. Institut d’Investigació i Innovació Parc Taulí (I3PT), Universitat Autònoma de Barcelona, Sabadell, Spain; 3 Computational and Clinical Nephrology. Parc Taulí Hospital Universitari. Institut d’Investigació i Innovació Parc Taulí (I3PT), Universitat Autònoma de Barcelona, Sabadell, Spain; 4 Grupo Mastertec, Barcelona, Spain; 5 Parc Taulí Hospital Universitari. Institut d’Investigació i Innovació Parc Taulí (I3PT), Universitat Autònoma de Barcelona, Sabadell, Spain; 6 CIBERes. Instituto de Salud Carlos III, Madrid, Spain

**Keywords:** 3d printing, Airway consumables, Personal protective equipment, Medical device, Impact analysis

## Abstract

**Background:**

To cope with shortages of equipment during the COVID-19 pandemic, we established a nonprofit end-to-end system to identify, validate, regulate, manufacture, and distribute 3D-printed medical equipment. Here we describe the local and global impact of this system.

**Methods:**

Together with critical care experts, we identified potentially lacking medical equipment and proposed solutions based on 3D printing. Validation was based on the ISO 13485 quality standard for the manufacturing of customized medical devices. We posted the design files for each device on our website together with their technical and printing specifications and created a supply chain so that hospitals from our region could request them. We analyzed the number/type of items, petitioners, manufacturers, and catalogue views.

**Results:**

Among 33 devices analyzed, 26 (78·8%) were validated. Of these, 23 (88·5%) were airway consumables and 3 (11·5%) were personal protective equipment. Orders came from 19 (76%) hospitals and 6 (24%) other healthcare institutions. Peak production was reached 10 days after the catalogue was published. A total of 22,135 items were manufactured by 59 companies in 18 sectors; 19,212 items were distributed to requesting sites during the busiest days of the pandemic. Our online catalogue was also viewed by 27,861 individuals from 113 countries.

**Conclusions:**

3D printing helped mitigate shortages of medical devices due to problems in the global supply chain.

**Supplementary Information:**

The online version contains supplementary material available at 10.1186/s12913-020-05891-2.

## Background

The SARS-CoV-2 pandemic overwhelmed healthcare systems, leaving many centers without essential equipment to prevent the spread of the virus or to treat COVID-19 patients [1]. Most of the products in short supply were consumables, such as connectors for invasive and noninvasive mechanical ventilation and personal protective equipment (PPE) [2, 3]. The companies that usually supply these products were unable to meet the high demand during the pandemic; many items were out of stock and inaccessible through the usual channels. The lack of equipment limited therapeutic options for many patients and contributed to the spread of the infection and collapse of the system by failing to protect healthcare professionals.

3D printing technology has been widely used in industrial companies around the world for years to create detailed models, customized parts, jigs and fixtures, end-use parts, concept models and visualization aids. Its rapid expansion is now revolutionizing some aspects of the medical field as well [4, 5]. The establishment of in-house specialized hospital-based 3D services has facilitated it. Nowadays, personal specific instruments (PSI) such as anatomical models and surgical guides, are used in the clinical practice [6, 7].

To cope with shortages and disrupted supply chains, various initiatives worldwide undertook to design and manufacture medical equipment with 3D printing in record time to supply hospitals [8, 9]. Designs for 3D printing have been made available online for many items, including airway consumables, facial masks and screens, and even entire ventilators [10, 11]. However, the impact of those initiatives has never been analyzed.

Our 3D surgical planning laboratory, a point-of-care 3D printing unit focused on personalized surgery, [7, 12] established a nonprofit integrated (end-to-end) solution to meet hospitals’ needs during the pandemic by supplying 3D-printed products. This solution included the identification, reengineering, validation, and supply of potentially lacking consumables based on our usual method of manufacturing customized medical devices in accordance with the ISO 13485 quality standard [13–15]. The initiative was co-managed with the College of Physicians and Surgeons of Barcelona (COMB) and the College of Industrial Engineers of Catalonia (EIC). To enable regional hospitals to request supplies, we created an online catalogue of 3D-printed consumables, including the files necessary to manufacture them so that any hospital worldwide with a 3D printing system could benefit.

To enable other regions to benefit from our experience, we describe the process of identifying, validating, regulating, and supplying 3D-printed medical equipment. We analyze the products identified as potentially lacking, the products validated, manufacturers’ characteristics, requests for products, distribution, and regulatory requirements, [16, 17] as well as the impact of this system during the peak of the SARS-CoV-2 pandemic in our region.

## Methods

### Identifying medical equipment in potential short supply

During the first 2 weeks of the pandemic in Spain (March 2020), our team worked with pulmonologists, intensive care physicians, and hospital purchasing managers to identify potentially unavailable medical equipment that could be manufactured using 3D printing. To determine the health system’s needs, we also considered news, other online information, and proposals from other health centers. Items that could not be validated in terms of knowledge or available facilities were excluded.

### Design

After the identification phase, two reengineering strategies were used to design items: 1) optical scanning and 2) de novo design referencing the dimensions of the original item.

For optical scanning, we used a hand-held 3D scanner (HandySCAN Black™ Elite, Creaform Inc.; Lévis, Canada) with dedicated software for reverse engineering and metrology (Geomagic® Design X, 3D Systems Inc.; Rock Hill, SC, USA or VxModel™, Creaform Inc.; Lévis, Canada). For de novo design, we used computer-aided design software (SOLIDWORKS®, Dassault Systèmes; Vélizy-Villacoublay, France).

When optical scanning could not detect specific areas of the product, we combined the two strategies.

All digital designs were exported as STL files (the most common file type for 3D printing).

### Validation system

Validation was based on the ISO13485 quality standard for customized medical device manufacturing [13, 15].

**Fig. 1:**
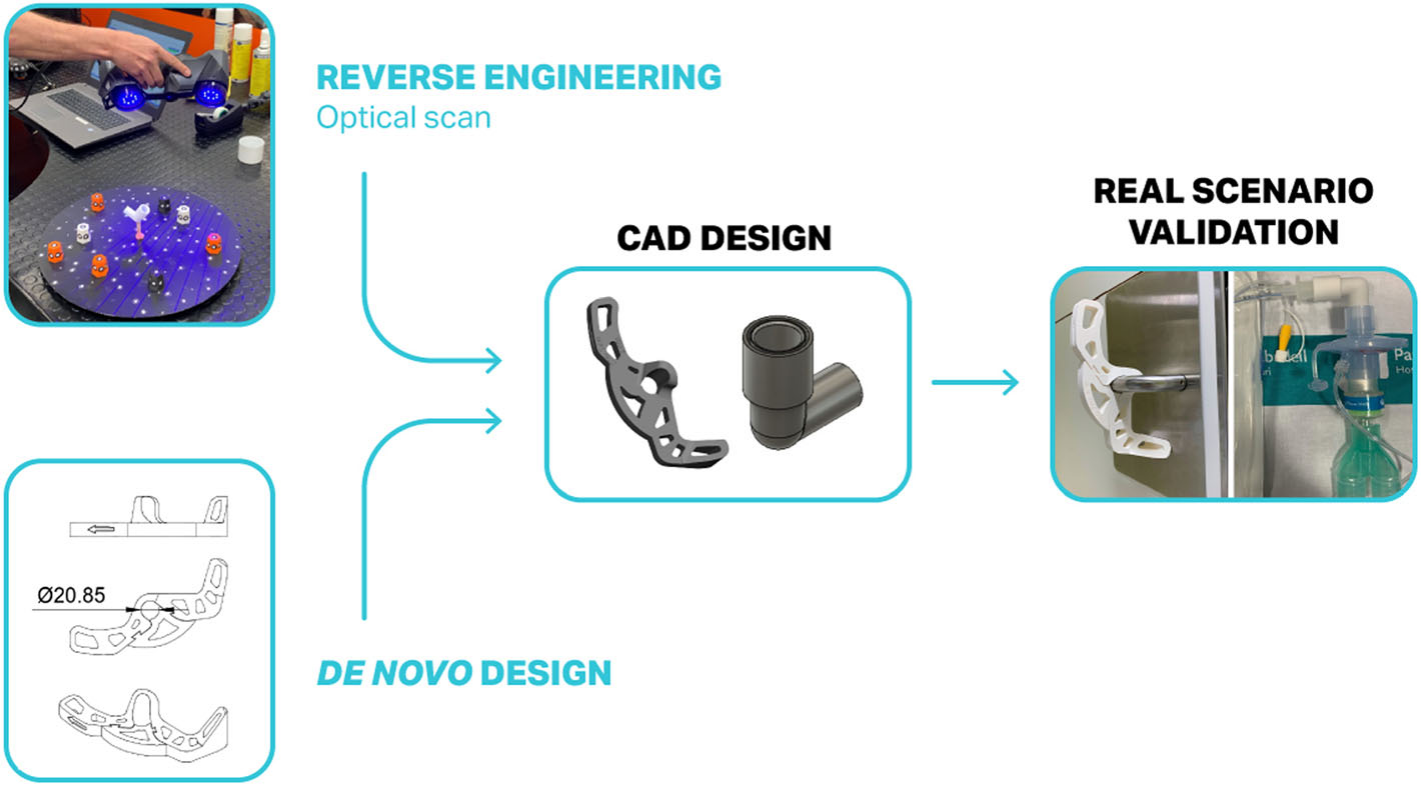
Prototype design approaches for producing 3D-printed medical devices

In the validation phase, we used 3D modeling software (Materialise 3-matic; Leuven, Belgium) for mesh analysis, optimization, and correction of the STL file. We printed small quantities of the items with polylactic acid on 3D printers (Ultimaker 3 or Ultimaker S5; Ultimaker B.V.; Utrecht, the Netherlands or MakerBot Replicator+; MakerBot Industries, LLC; New York, NY, USA), ensuring the dimensions of 3D-printed pieces with certified vernier calipers. Afterward, pieces were tested in real clinical scenarios (Fig. 1). Devices that needed additional considerations or calibrations (3DPT21, 3DPT22, 3DPT23, and 3DPT33: see the Additional file 1) were tested in an experimental model using a polygraph (PowerLab 16 SP; ADInstruments, Dunedin, NZ) equipped with an S300 pneumotachograph (dead space, 80 ml) and a pressure transducer [18].

Once the design was validated, a technical documentation form was completed for each item, including information regarding its use, technical design, and manufacturing specifications as well as schematic diagrams and pictures of the products.

To ensure the items’ functionality, we asked manufacturers to supply units for testing made with different 3D-printing technologies and different materials (only biocompatible materials fulfilling the ISO 10993 / USP class VI quality standards for at least skin and mucous contact).

Specific cleaning procedures were defined for each material to prevent thermal deformation and alteration of chemical properties.

### Online catalogue

Validated items were included with their STL files and technical documentation in an online catalogue on our institution’s open website [19].

### Local manufacture and distribution

**Fig. 2:**
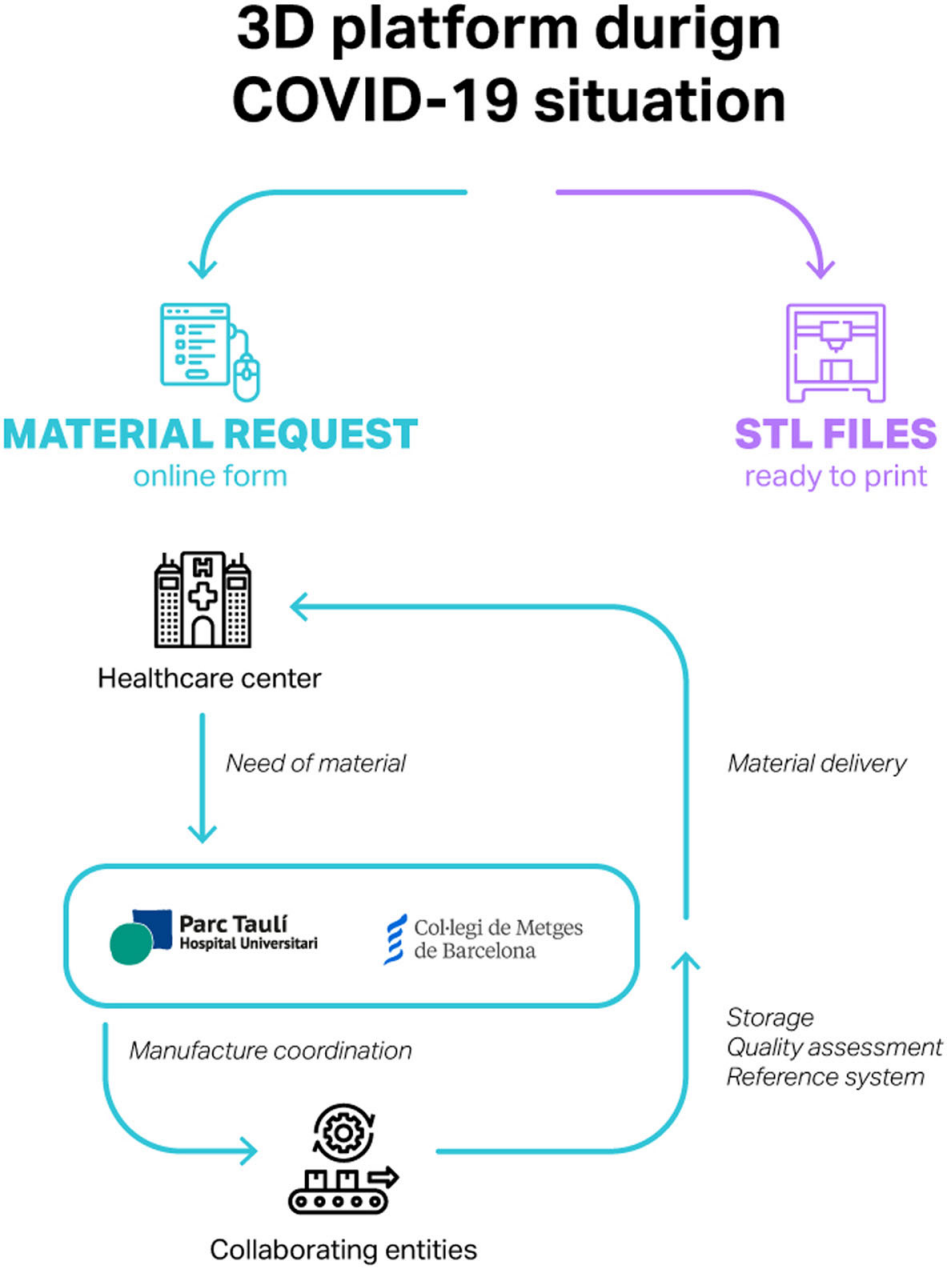
Scheme of the organization of the system established to supply hospitals in Catalonia with 3D-printed medical equipment

In parallel with the publication of the catalogue, we designed a system to supply the items to health centers in our region. Orders were placed through a form on our institutional website [20] and transmitted to COMB and EIC, who coordinated the manufacture, storage, and distribution (Fig. 2).

Manufacturing companies from different sectors willing to collaborate in the context of the COVID-19 crisis were grouped and organized through a citizens’ initiative platform [21]. Companies received no financial compensation for these actions.

Items for which full details about manufacture were unavailable were excluded from centralized distribution channels. All items were washed, disinfected, and dried before distribution.

### Regulatory requirements

The 3D-printed items produced are consumables or disposable components necessary for the proper function of electromedical equipment, but not considered critical by manufacturers. Given the manufacturers’ inability to supply these products during the COVID-19 pandemic health emergency, European in-house manufacturing regulations allow them to be manufactured as custom medical devices with appropriate safety guarantees by 3D printing for hospitals’ exclusive use, waiving the need for CE marking [22] and assigning the responsibility for the use of these products to the health centers. Hospital executives accepted the responsibility for using the products.

### Impact measurements

Data regarding the number of individual visitors and the total number of visits to the catalogue were extracted from Google Analytics.

The number of deaths due to COVID19 and number of infections in each Spanish region were extracted from the Spanish Ministry of Health’s website [23].

Information regarding orders, manufacturers, and 3D-printed items was collected in a dedicated database.

### Statistical analysis

We compiled descriptive statistics, reporting frequencies and percentages. To evaluate the relationship between visits to the online catalogue and incidence of COVID-19 in Spain, we used Pearson correlation, considering *P* < 0·05 significant.

## Results

A total of 33 necessary items that were potentially lacking during the COVID-19 (March–April 2020) were identified; 26 (78·8%) of these were validated and included in the online catalogue [23/26 (88·5%) components for invasive or noninvasive ventilation and 3 (11·5%) PPE items (“hands-free” doorknobs, protective visors, and ear protectors for masks)]. Other PPE items (e.g., facemasks) could not be validated, as we could not guarantee their safety. Table S1 presents the complete catalogue published online.

Our hospital’s consumption of specific products allowed us to gauge demands at other centers. We received orders for 15,699 items from 25 health centers in the region [19 (76%) hospitals, 2 (8%) primary care centers, and 4 (16%) nursing homes]. A total of 19,212 items were distributed to the health centers that ordered them. The difference between the number of items ordered through the website and the number distributed (3513) is due to some hospitals reordering directly from COMB after receiving the first order submitted through the website.

A total of 60 companies and 10 manufacturers associations from a diverse range of sectors participated in production (Table 1). Companies specialized in 3D technology provided the most units (*n* = 5234; 23·6%), followed by automotive companies (*n* = 2854; 12·9%). The 10 manufacturers associations used non-industrial 3D printers to manufacture 1847 (8·3%) items; these associations produced only PPE items that did not require such a thorough validation process. Nearly half the companies (*n* = 25; 42·4%) had more than 250 employees; 7 (11·9%) had from 51 to 249 employees, 13 (22%) had from 11 to 49 employees, and 14 (23·7%) had 10 employees or fewer. Nearly half of all companies 27 (45%) had international import or export relations or international headquarters or branches.

**Table 1:** Companies involved in the manufacture of 3D medical devices, number of items and number of companies per industry type

Company Type	3D printed units N (%)	number of companies N (%)
3D-printing Industry	5234 (23·6%)	13 (18·6%)
Automotive Industry	2854 (12·9%)	3 (4·3%)
Dental Industry	2641 (11·9%)	9 (12·9%)
Manufacturers associations	1847 (8·3%)	10 (14·3%)
Universities	1579 (7·1%)	3 (4·3%)
Chemical Industry	1483 (6·7%)	2 (2·9%)
Naval and railway Industry	1193 (5·4%)	1 (1·4%)
Plastic Industry	1053 (4·7%)	4 (5·7%)
Engineering Consulting	781 (3·5%)	6 (8·6%)
Electric Industry	686 (3·1%)	4 (5·7%)
Metal Industry	473 (2·1%)	3 (4·3%)
Biomedical Industry	469 (2·1%)	1 (1·4%)
Jewelry	403 (1·8%)	1 (1·4%)
Social Organizations	353 (1·6%)	3 (4·3%)
Medical Industry	284 (1·3%)	1 (1·4%)
Food Industry	284 (1·3%)	2 (2·9%)
Packaging Industry	240 (1·1%)	2 (2·9%)
Medical Center	69 (0·3%)	1 (1·4%)
Unknown	267 (1·2%)	1 (1·4%)
TOTAL	**22,193 (100%)**	**70 (100%)**

Table S2 provides information about the types of devices and material used to manufacture them.

**Fig. 3:**
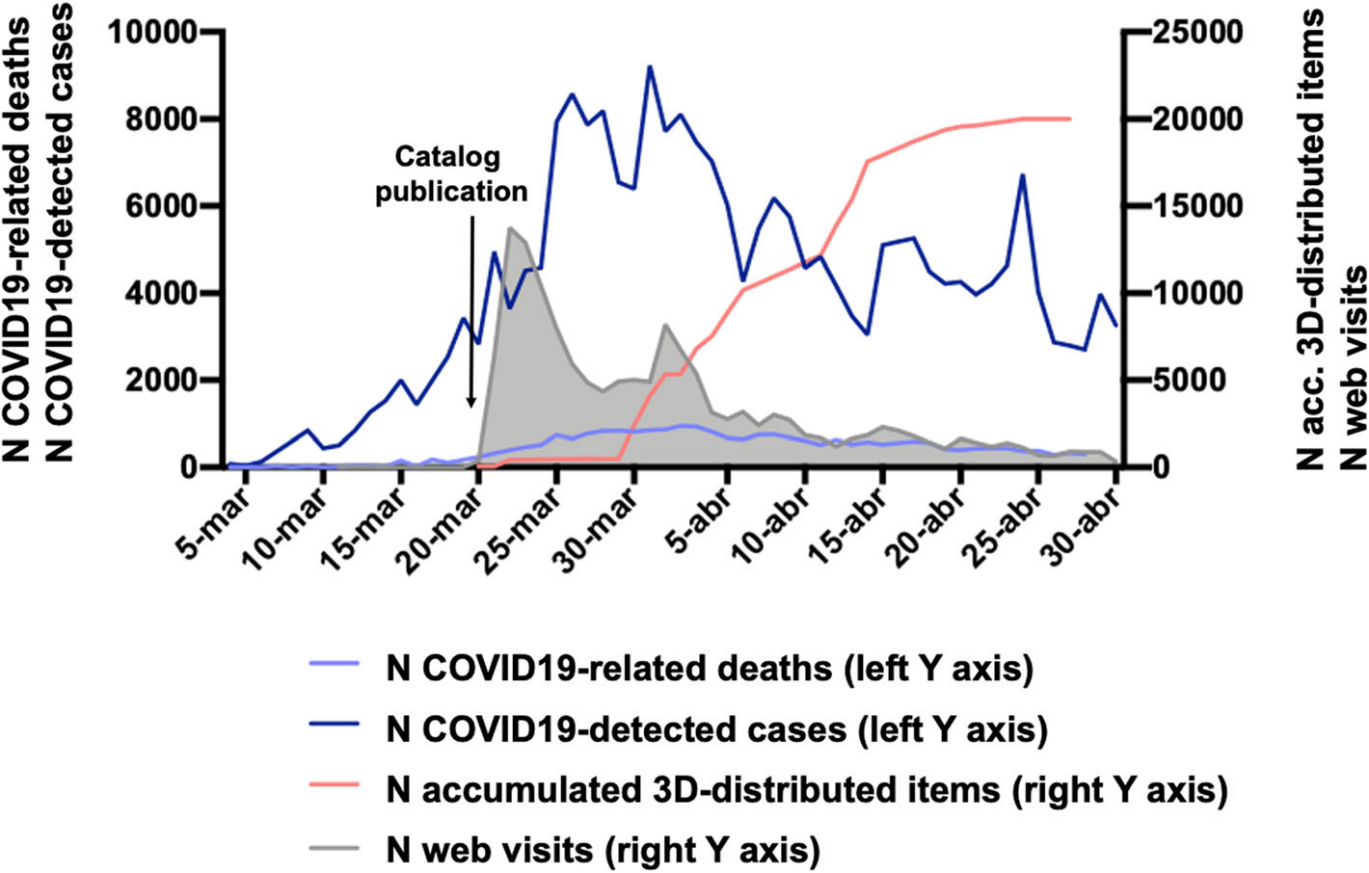
Timeline showing the numbers of COVID-19-related deaths, COVID-19 cases detected, web visits, and accumulated 3D-manufactured items

To determine when interest in 3D-printed projects was greatest, we analyzed the dates of visits to the online catalogue according to the number of hospitalizations for COVID-19. Most visits took place during the expansion phase of the epidemic in Spain (Fig. 3). The number of visits increased again at the peak of the country’s epidemic and gradually decreased thereafter as the number of new cases decreased. At the peak, 21% of all the items produced had been delivered, although orders and deliveries continued through late April. Thus, before the peak, hospitals were already seeking alternative sources of medical equipment, anticipating or even already suffering shortages, and 3D printing provided a fast, reliable solution.

**Fig. 4:**
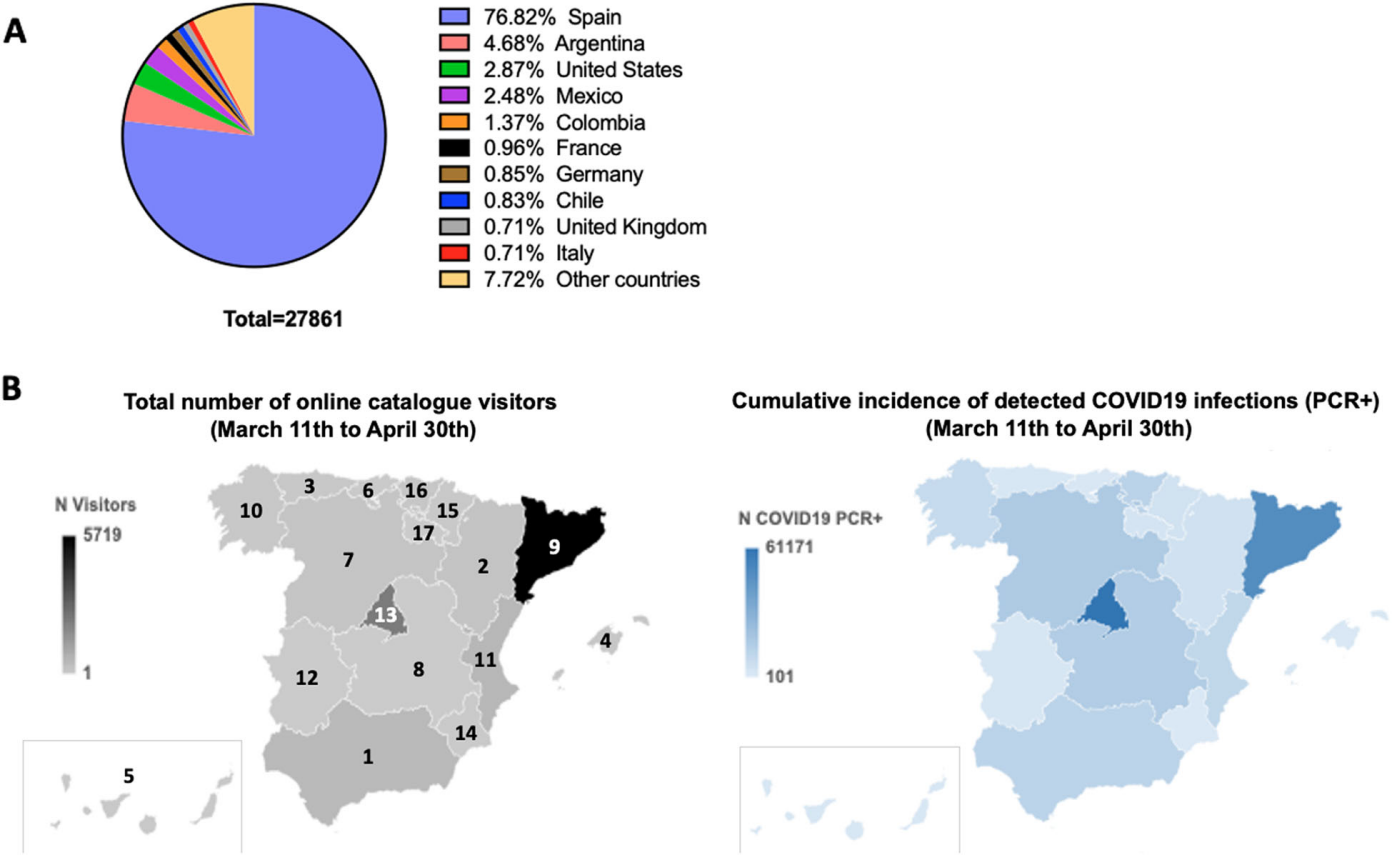
**a** Top 10 countries with the most visitors to the website www.tauli.cat/institut/. **b** Spain Autonomous Comunities maps showing the total number of visis to the online catalogue (left) and the number of COVID-19 detected cases (right). Autonomous Comunities are indicated by numbers: 1. Andalucia, 2. Aragon, 3. Asturias, 4. Balearic Islands, 5. Canary Islands, 6. Cantabria, 7. Castile and León, 8. Castile-La Mancha, 9. Catalonia, 10. Galicia, 11. Valencia, 12. Extremadura, 13. Madrid, 14. Murcia, 15. Navarre, 16. Basque Country, 17. La Rioja. The maps depicted in Fig. 4 were created by the authors of this manuscript

From March 11 through April 30, 2020, our online catalogue of validated 3D-printed medical equipment received 58,904 views from 27,861 unique users from 113 countries. Due to proximity and outreach effects, most (76%) visitors were from Spain; however, the countries with the most visitors also included distant lands such as Argentina (4·68%), the USA (2·87%), Mexico (2·48%), and Colombia (1·37%) (Fig. 4a).

We analyzed the distribution of visitors from different regions of Spain. Most visitors were from Catalonia, where our center and the distribution network are located, and Madrid, the two Autonomous Communities with the highest incidence of COVID-19. Pearson correlation coefficient (r) showed a strong positive linear association (*r* = 0·71; *P* < 0·001) between the total number of visits to the online catalogue and the cumulative incidence of SARS-CoV-2 infection in the different regions of Spain (Fig. 4b).

## Discussion

The speed, availability of material, and maneuverability of 3D printing made it useful in mitigating the shortage of medical equipment during the emergency situation generated by the SARS-CoV-2 pandemic.

Numerous initiatives worldwide have proved the usefulness of this technology, sharing designs for potentially lacking products online that can be manufactured by 3D printing (e.g., facemasks and connectors for snorkeling masks to be used with noninvasive ventilators). Yet, at least in the first stages of the pandemic, it was difficult to find a reliable repository of 3D-printer-manufactured products whose clinical utility was validated according to current regulatory legislation. For this reason, covered by in-house manufacturing regulations, we focused our efforts on validating similar but reliable medical devices and distributing them to hospitals on request [24].

One important factor in our success was our pre-existing 3D laboratory, created by physicians at our referral hospital to enable point-of-care 3D printing for personalized medicine. Another key factor for our success was the decision to supply other hospitals in the area through a network of collaborators that included official organisms (COMB and EIC) as well as a wide range of committed companies with the facilities required to manufacture the items in our catalogue in record time without seeking financial gain. Our experience shows that any kind of industry with 3D printers, regardless of their usual sphere of activity, can help in emergencies, provided the system has a good organizational strategy with well-defined steps for designing, manufacturing, and distributing items made under regulation.

One of our major challenges for developing and maintaining the presented system was to identify items in potential short supply. Also, the organization of the whole system had to be designed very accurately to be effective. Finding companies with 3D printing facilities to manufacture the items was not an issue, nor even the production costs, since many industries volunteered, and it was a non-profit making scheme. In order for this system to be successful in other countries, the organization has to be adapted accordingly, coordinating local authorities and industries, profession associations, social media, etc.

Despite emerging as a valid solution in this emergency situation, 3D printing is not the preferred method for manufacturing PPE under normal conditions (excluding the prototyping stages). Traditional manufacturing may take months, not only to create, but also to transfer the final products to the end-users. 3D printing can overcome these limitations; you can produce items on-site. However, the number of items manufactured by 3D printing is not as large as and in traditional manufacturing, since they can only be printed in small amounts at the same time. Overall, 3D printing is still a considerably expensive investment compared to traditional manufacturing machines for large production volumes [24].

On the other hand, and on a positive note, 3D printing allowed us to customize some of the items, for example 3DPT012 (see the Additional file 1). Given that COVID-19 patients needed more oxygen flow than a conventional patient, this connector for continuous positive airway pressure (CPAP) was adapted by increasing the diameter of the side tube. This allowed patient recovery to improve quicker.

Among the limitations of our approach was the inability of additive manufacturing to fulfill all the requirements for the production of validated devices. For instance, limited availability of flexible biocompatible material reduced the scalability of production, making it impossible to meet the demand for some products (e.g., 3DPT006; see the Additional file 1). Additionally, despite their viability in laboratory simulations, certain consumables (3DPT021, 3DPT022, 3DPT023, 3DPT033; see the Additional file 1) had yet to be certified for clinical use by the Spanish regulatory agency (Spanish Agency of Medicines and Medical Devices (AEMPS)) when the pandemic peaked.

Moreover, the outsourcing of the manufacture of medical supplies from outside our country hampered our ability to react to the pandemic by stepping up certified production. Local companies that had adopted 3D printing for other purposes were able to convert their production to compensate in part for the lack of dedicated medical supplies industry.

Our analysis focuses only on the hospitals that ordered medical items through our channel, although other hospitals outside the distribution area or companies with the means to manufacture items could also benefit from the designs without using the established channel. The high number of views from 113 countries suggests that this might have happened in hospitals all over the world. Undoubtedly, 3D printing has now become a quite affordable, user-friendly, and widely available technology that could be worth investing in in places all over the world, irrespective of the country’s economic status.

## Conclusions

This work provides evidence that technological advances, such as 3D printing, have many useful applications, also during emergency situations. It has empowered communities to fight against the pandemic of SARS-CoV-2 and has benefited hospitals immensely by manufacturing medical equipment, thus mitigating shortages. Numerous regions worldwide can benefit from this approach, also to prevent or overcome shortages of medical equipment.

## Supplementary Information

**Additional file 1:** **Table S1.** Catalogue of validated 3D-printed medical devices. **Table S2.** Number, type, and material of items manufactured by 3D printing.

## Data Availability

The datasets used and/or analyzed during the current study are available from the corresponding author on reasonable request.

## Abbreviations

3D: Three-dimensional
AEMPS: Spanish Agency of Medicines and Medical Devices
CE marking: European Conformity marking
COMB: College of Physicians and Surgeons of Barcelona
CPAP: Continuous positive airway pressure
EIC: College of Industrial Engineers of Catalonia
PPE: Personal protective equipment
SARS-CoV-2: Severe acute respiratory syndrome coronavirus 2
STL files: File format native to the stereolithography CAD software
