# Supplementary material for: Integrated 3D printing solution to mitigate shortages of airway consumables and personal protective equipment during the COVID-19 pandemic
Source: BMC Health Serv Res. 2020 Nov 12;20:1035. doi: 10.1186/s12913-020-05891-2 (PMC7657712; doi:10.1186/s12913-020-05891-2)
Supplement: Supplementary file 1 — Additional file 1: Table S1. Catalogue of validated 3D-printed medical devices. Table S2. Number, type, and material of items manufactured by 3D printing. [file 12913_2020_5891_MOESM1_ESM.pdf]

Additional file 1

Table of contents

1.- Table S1.- Catalogue of validated 3D-printed medical devices............................2

2.- Table S2.- Number, type, and material of items manufactured by 3D printing ...11

1.- Table S1.- Catalogue of validated 3D-printed medical devices

Category	Virtual Model	3D-printed unit
Airway connectors and replacements	3DPT001	
	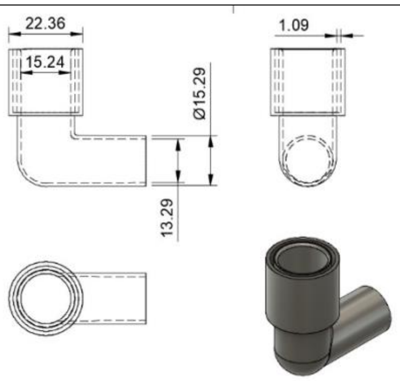	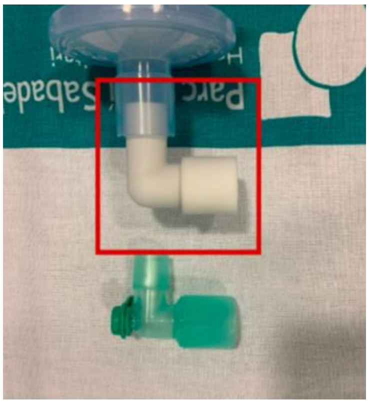
	3DPT005	
	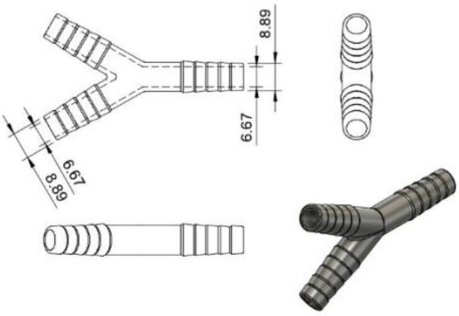	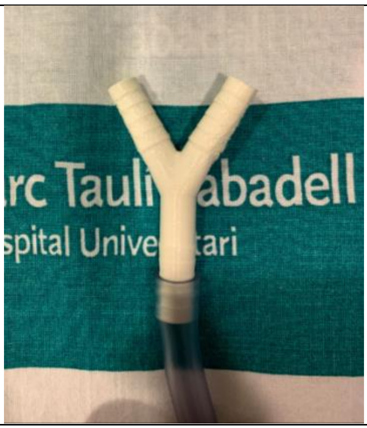
	3DPT006	
	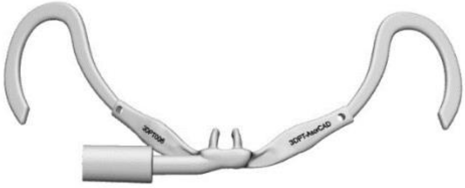	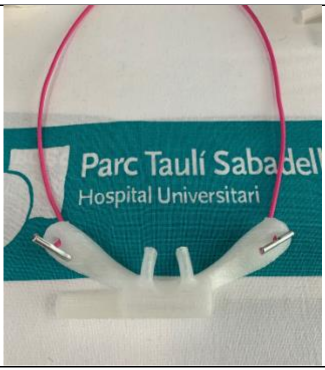
	3DPT007	
	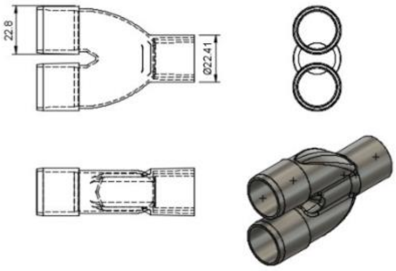	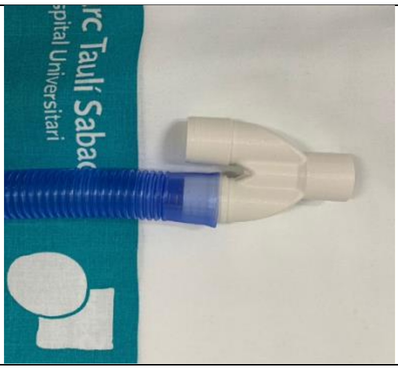

<table>
<tr><td rowspan="7">Airway connectors and replacements</td><td colspan="2">3DPT008</td></tr>
<tr><td>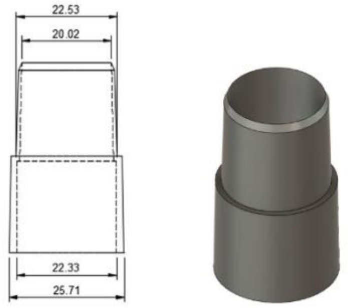
</td><td>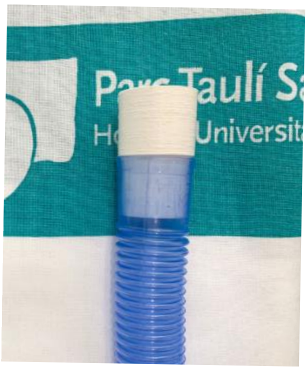</td></tr>
<tr><td colspan="2">3DPT009</td></tr>
<tr><td>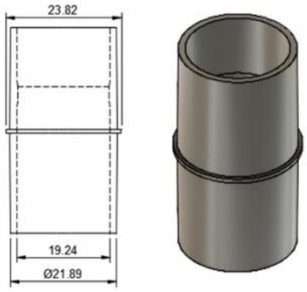
</td><td>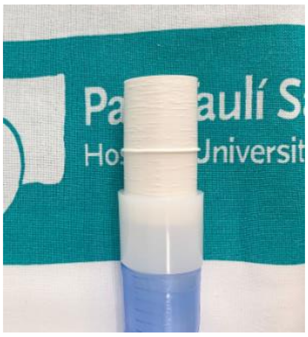</td></tr>
<tr><td colspan="2">3DPT010</td></tr>
<tr><td>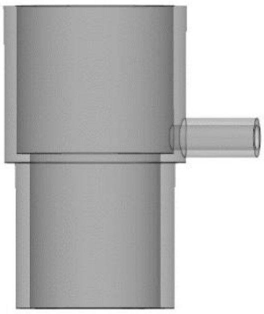</td><td>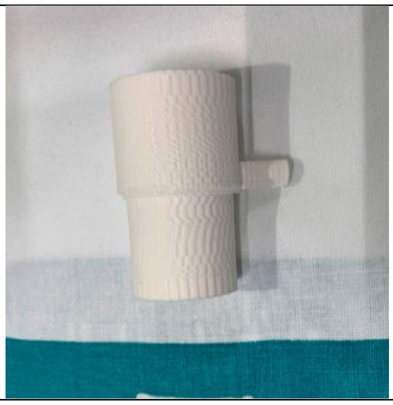</td></tr>
<tr><td colspan="2">3DPT011</td></tr>
</table>

	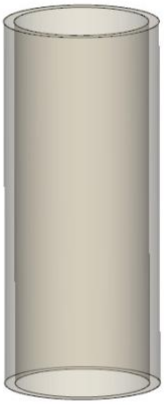	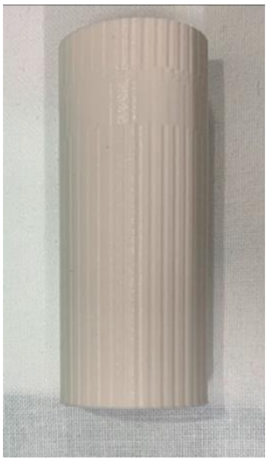

<table>
<tr><td rowspan="8">Airway connectors and replacements</td><td colspan="2">3DPT012</td></tr>
<tr><td>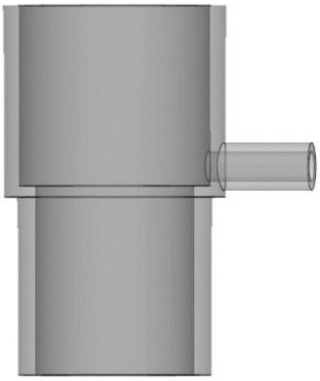</td><td>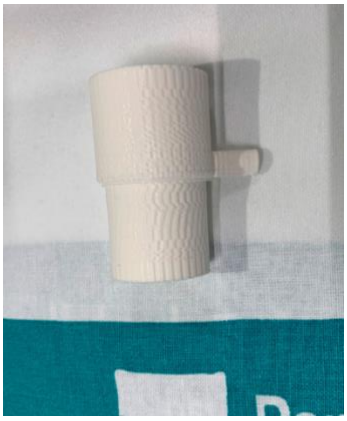</td></tr>
<tr><td colspan="2">3DPT013</td></tr>
<tr><td>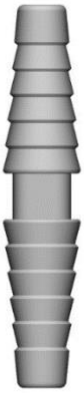</td><td>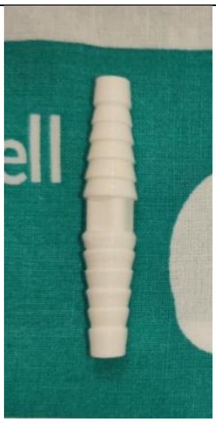</td></tr>
<tr><td colspan="2">3DPT014</td></tr>
<tr><td>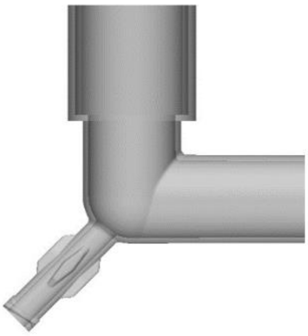</td><td>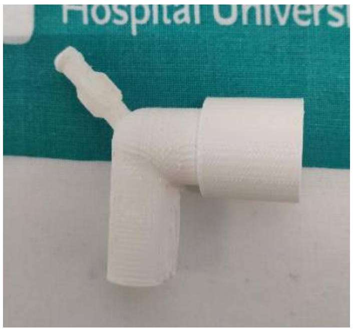</td></tr>
<tr><td colspan="2">3DPT015</td></tr>
<tr><td>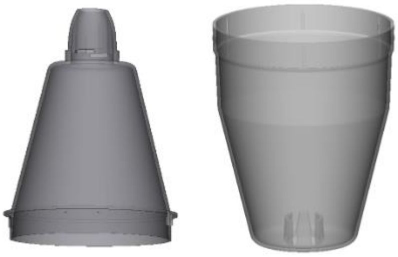</td><td>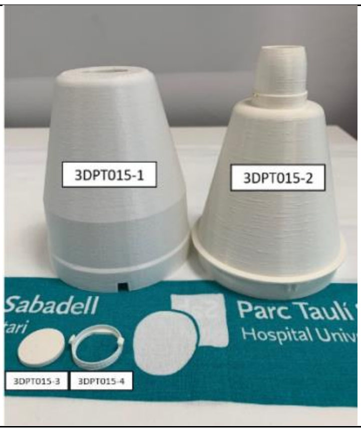
</td></tr>
</table>

<table>
<tr><td rowspan="6">Airway connectors and replacements</td><td colspan="2">3DPT016</td></tr>
<tr><td>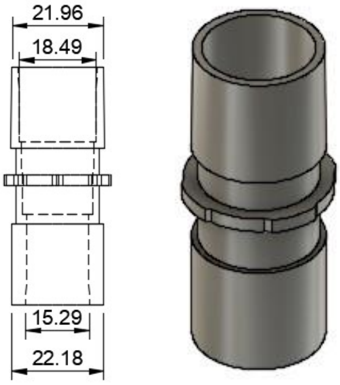
</td><td>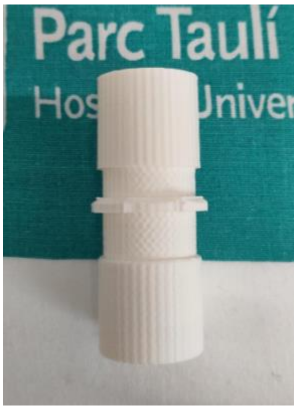
</td></tr>
<tr><td colspan="2">3DPT018</td></tr>
<tr><td>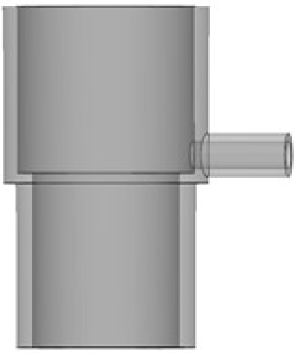</td><td>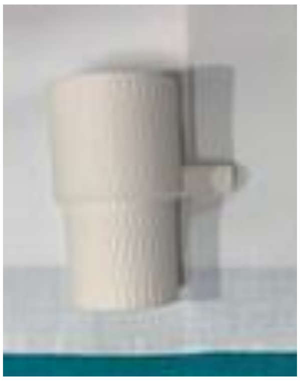</td></tr>
<tr><td colspan="2">3DP019</td></tr>
<tr><td>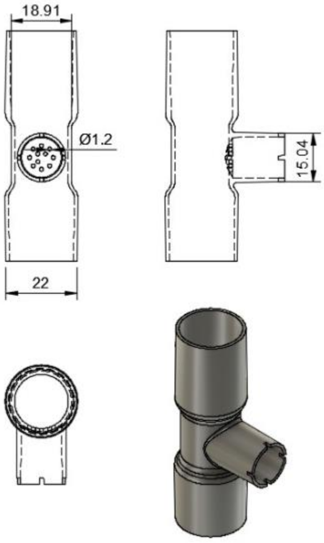
</td><td>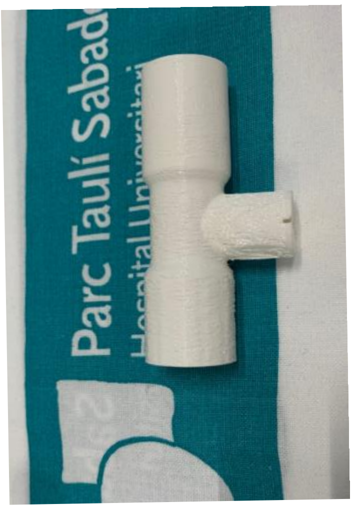</td></tr>
</table>

<table>
<tr><td rowspan="8">Airway connectors and replacements</td><td colspan="2">3DPT022</td></tr>
<tr><td>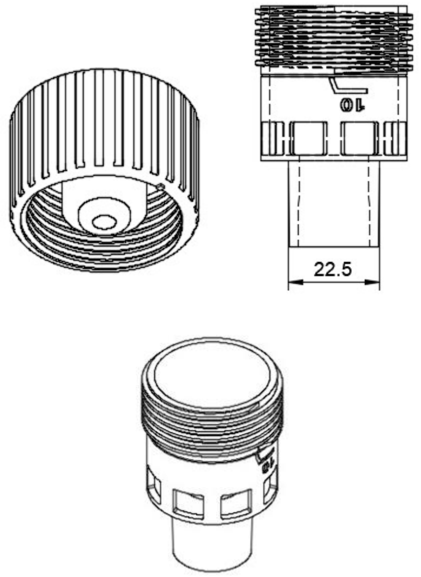
</td><td>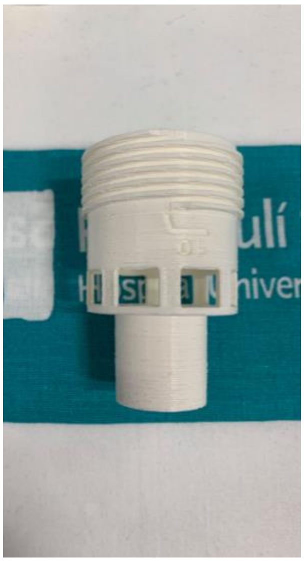</td></tr>
<tr><td colspan="2">3DPT024</td></tr>
<tr><td>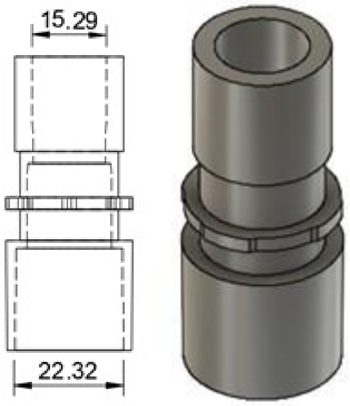
</td><td>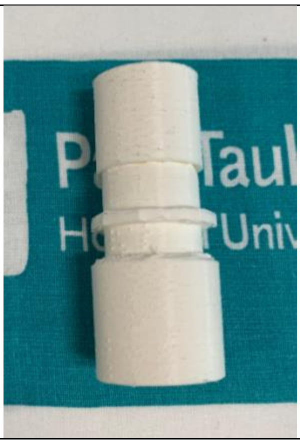</td></tr>
<tr><td colspan="2">3DPT025</td></tr>
<tr><td>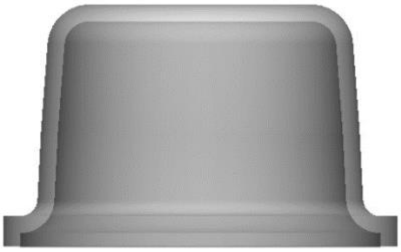</td><td>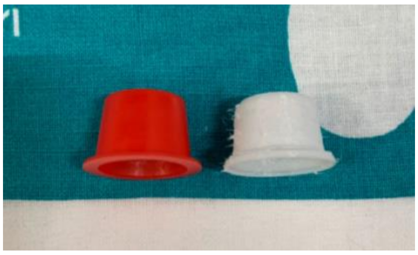</td></tr>
<tr><td colspan="2">3DPT026</td></tr>
<tr><td>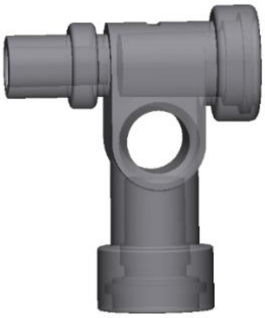</td><td>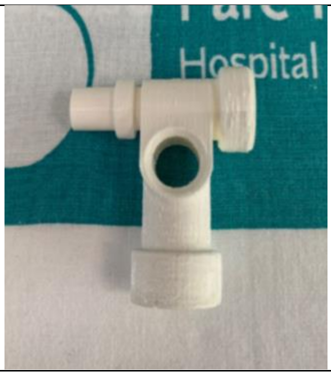
</td></tr>
</table>

	3DPT027	
	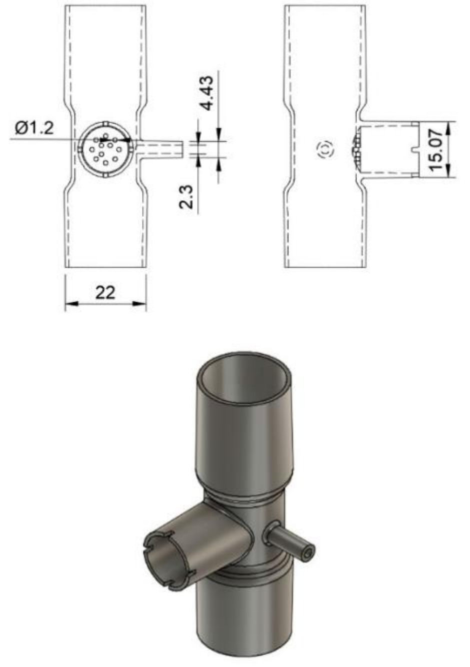	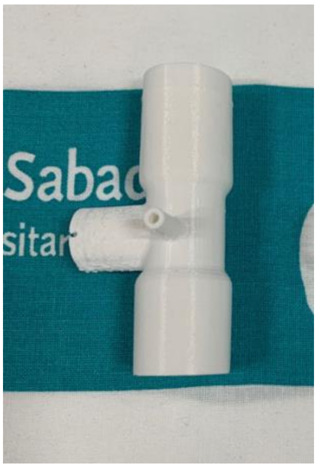
Protection and Prevention Systems	3DPT002	
	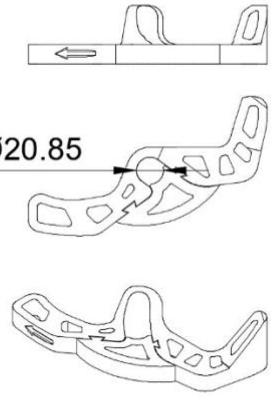	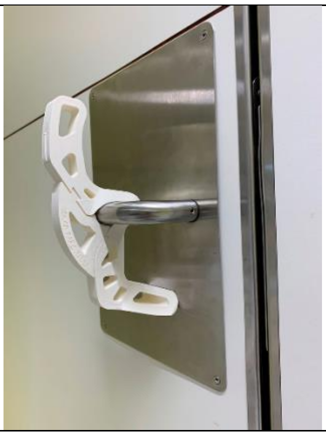
	3DPT020-HSJ	
	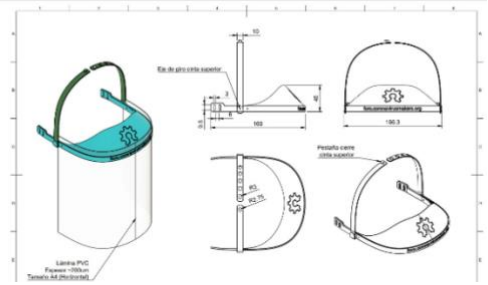	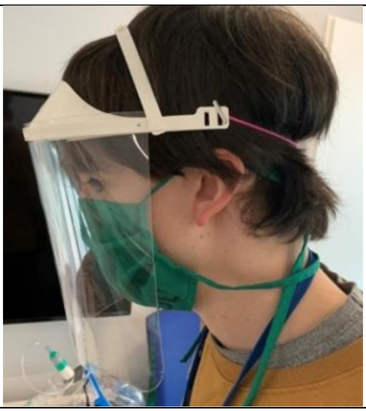

	3DPT031	
	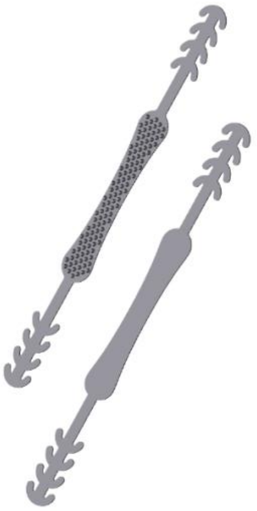	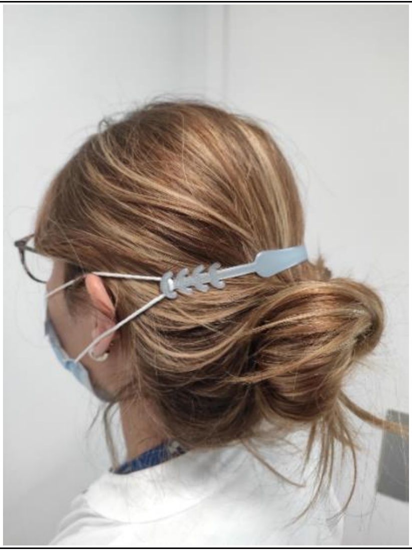
Non-invasive Ventilation	3DPT021 (including 3DPT021-1 and 3DPT021-2)	
	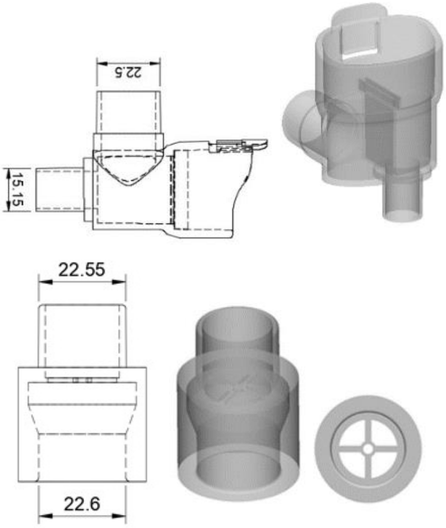	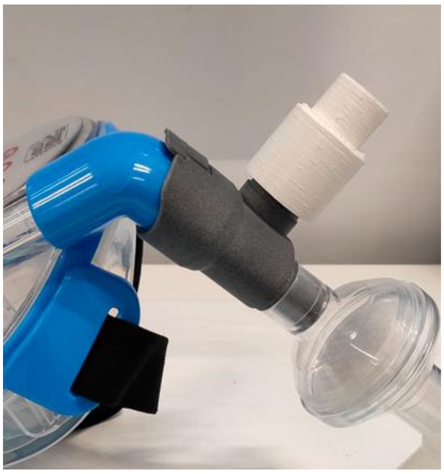
	3DPT023 (including 3DPT023-1 and 3DPT023-2)	
	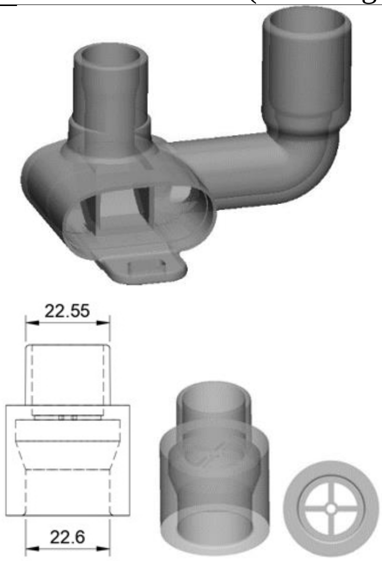	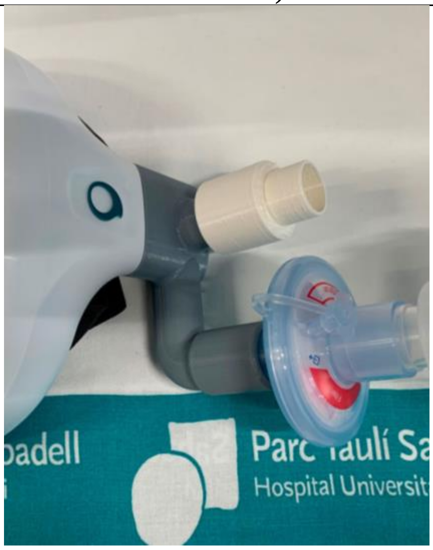

	3DPT033 (including 3DPT033-1 and 3DPT033-2)	
	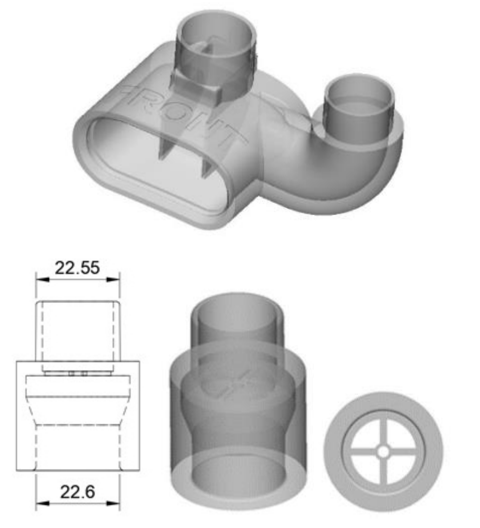	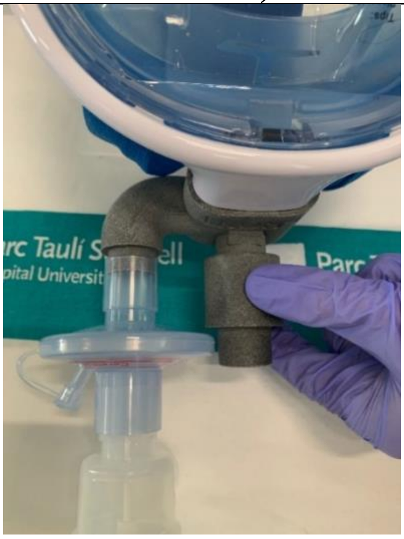

2.- Table S2.-Number, type, and material of items manufactured by 3D printing.

MATERIALS

ID	PA12	PA2200	Dental Photoreactive Resin	ABS Medical	PET-G	PC	PLA	Other Biocompatible Materials	Unknown materials or Origin	TOTAL
**3DPT001**	767	44	66	82	44	-	323	23	112	**1461**
**3DPT002**	1	-	-	-	-	-	161	-	-	**162**
**3DPT005**	332	-	346	395	148	-	320	17	95	**1653**
**3DPT006**	2	-	2	-	4	-	-	217	4	**229**
**3DPT007**	319	-	367	339	67	-	639	14	187	**1932**
**3DPT008**	243	-	186	133	2	30	1121	249	696	**2660**
**3DPT009**	240	-	151	39	2	50	1248	26	463	**2219**
**3DPT010**	146	-	70	53	3	-	202	26	31	**531**
**3DPT011**	178	-	113	-	3	30	93	19	7	**443**
**3DPT012**	143	-	47	73	18	-	134	29	20	**464**
**3DPT013**	626	-	259	1174	6	-	410	37	37	**2549**
**3DPT014**	165	-	86	-	2	-	177	30	44	**504**
**3DPT016**	11	-	156	108	1	-	201	130	29	**636**
**3DPT018**	21	-	-	114	5	-	89	244	43	**516**
**3DPT019**	42	-	8	102	39	-	347	269	100	**907**
**3DPT020**	1	-	-	-	-	-	187	-	-	**188**
**3DPT021-1**	185	-	65	23	-	-	21	75	-	**369**
**3DPT021-2**	239	-	67	31	133	-	72	68	66	**676**
**3DPT022**	-	-	-	20	-	-	10	-	-	**30**
**3DPT023-1**	-	-	-	-	-	-	10	-	-	**10**
**3DPT023-2**	-	-	-	-	-	-	10	-	-	**10**
**3DPT024**	24	-	2	69	-	-	183	104	41	**423**
**3DPT025**	2	-	43	-	294	-	-	1042	20	**1401**
**3DPT026**	-	61	-	-	2	-	36	43	-	**142**
**3DPT027**	-	-	-	-	-	-	85	-	-	**85**
**3DPT029**	-	-	4	-	-	-	-	-	-	**4**
**3DPT031**	-	-	-	-	-	-	1841	-	-	**1841**
**3DPT033**	4	-	-	-	-	-	4	-	-	**8**

**Others**	-	-	-	-	-	30	-	-	110	**140**
**TOTAL**	**3691**	**105**	**2038**	**2755**	**773**	**140**	**7924**	**2662**	**2105**	**22193**

Abbreviations: PA12, Polyamide 12; PA2200, Polyamide 2200; ABS, Acrylonitrile butadiene styrene; PET-G, Polyethylene terephthalate glycol; PC, Polycarbonate; PLA, Polylactic acid.
